# Imparting Photocatalytic and Antioxidant Properties to Electrospun Poly(L-lactide-*co*-D,L-lactide) Materials

**DOI:** 10.3390/polym16131814

**Published:** 2024-06-26

**Authors:** Ina Anastasova, Petya Tsekova, Milena Ignatova, Olya Stoilova

**Affiliations:** Laboratory of Bioactive Polymers, Institute of Polymers, Bulgarian Academy of Sciences, Akad. G. Bonchev St., bl. 103A, 1113 Sofia, Bulgaria

**Keywords:** electrospinning, electrospraying, PLDLLA, Fe_3_O_4_, ZnO, antioxidant activity, photocatalytic activity

## Abstract

The focus of the present study is on the fabrication of effective and eco-friendly hybrid electrospun materials based on poly(L-lactide-*co*-D,L-lactide) (PLDLLA), Fe_3_O_4_ and ZnO with an appropriate design for antioxidant and photocatalytic performance. The design of the fibrous materials was purposely tailored in one step by electrospinning and simultaneous electrospinning/electrospraying. Electrospinning of PLDLLA and its mixture with Fe_3_O_4_ resulted in the fabrication of materials with design type “*in*”. Furthermore, the surface of the electrospun PLDLLA and Fe_3_O_4_-*in*-PLDLLA was decorated with ZnO particles by simultaneous electrospraying, thus materials with design type “*on*” were obtained. In this case, quaternized *N*,*N*,*N*-trimethyl chitosan iodide (QCOS) was used as a sticking agent of ZnO particles onto the fiber’s surface. Different structures and morphologies of the electrospun materials were observed by SEM equipped with EDX and TEM. TGA and XRD analyses show that the presence of inorganic particles had an impact on the thermal properties and crystallinity of the electrospun materials. Furthermore, the material type “*on*” showed improved wettability with a water contact angle less than 90° compared to the material type “*in*” with an angle larger than 90°. In particular, the presence of Fe_3_O_4_ imparts complementary magnetic properties, while ZnO considerably increased the antioxidant activity of the fibrous materials. Materials with design type “*on*” displayed over 70% radical scavenging capacity in contrast to the material type “*in*” with less than 20% capacity within 30 min of contact. Moreover, the purposely tailored design type “*on*” materials provided excellent photocatalytic degradation of model organic pollutant methylene blue dye under UV light irradiation even after 5-fold use, and at the end of the fifth cycle these materials degraded more than 90% of the dye. These results reveal not only a strategy for the fabrication of electrospun hybrid bio-based materials with targeted design but also provide a promising, simple and effective way for mitigating water pollution.

## 1. Introduction

In recent decades, water pollution by various organic pollutants and dyes used in the paper, food, textile and cosmetic industries has grown to be a major worldwide issue. Various conventional methods are used for the treatment of polluted waters, such as adsorption, sedimentation, filtration, biological treatments, and chemical oxidation [1]. They are costly and have several limitations, such as the need for pre-treatment of wastewater, limited practicability, poor efficacy and secondary contamination [1,2,3]. In order to address current needs in water purification from organic pollutants and dyes, heterogeneous photocatalysis seems to be very promising, eco-friendly and effective [4,5,6]. The advantages of this technology are that it efficiently removes these pollutants under standard temperature and pressure, does not produce secondary pollution, and at the end of the process, the photocatalyst can be recycled. Moreover, in this process, the organic molecules might be completely oxidized to water and CO_2_ [7,8,9].

Among heterogeneous photocatalysts, zinc oxide (ZnO) is known as inexpensive, biocompatible and with low toxicity, as well as with good optical properties and antimicrobial activity against common pathogens [10,11,12]. In addition, much of the literature describes in detail its photocatalytic mechanism towards various organic pollutants under UV irradiation [13,14,15]. Notably, ZnO had higher photocatalytic efficiency than TiO_2_ due to its better UV-absorption ability, thus degradation of organic pollutants does not depend on the pH of the medium. Despite these promising properties, freely suspended ZnO particles are difficult to separate from the reaction medium, leading to secondary pollution. Therefore, to avoid this difficulty in their removal, ZnO particles are incorporated in various polymer matrices. In particular, fibrous polymer materials with embedded ZnO particles made by electrospinning have attracted considerable attention, especially in the field of water remediation, mainly due to their controllable morphology with a high surface area-to-volume ratio, lightness and ease of handling. To date, different synthetic polymers such as polyacrylonitrile, poly(vinyl alcohol), polyamide, polyethylene oxide, polyacrylic acid and polyimide have been successfully used as matrices for electrospinning with ZnO [16,17,18,19,20,21,22,23]. It was shown that these electrospun hybrid materials combine beneficial properties of ZnO and possess excellent filtration performance. However, the ZnO particles are mainly distributed inside the fibers, which prevents them from actively participating in the photocatalytic reactions and reduces their activity. Also, taking into account the toxic accumulation of plastics in the environment, the use of bio-based and biodegradable polymeric materials is extremely imperative, instead of the aforementioned synthetic polymers.

In terms of water purification, iron oxide (Fe_3_O_4_) particles are widely used for easy removal from the reaction medium. These particles emerge as prominent materials due to their unique features, such as tunable magnetic properties, adsorption capacity, excellent biocompatibility and low toxicity. In our previous studies, we have shown the successful fabrication of electrospun multifunctional materials based on poly(3-hydroxybutyrate) (PHB), TiO_2_ and Fe_3_O_4_ [24,25,26], as well as based on poly(L-lactide) (PLA) and ZnO [27,28]. The results demonstrated that the incorporated in the fiber Fe_3_O_4_ retained its magnetic properties and was sufficient for easy removal of the electrospun materials from the reaction medium by application of the external magnetic field. In addition, the obtained hybrid fibrous materials demonstrated excellent photocatalytic and antimicrobial activity. Nevertheless, for biodegradability, PHB is stiff and brittle, while PLA is semi-crystalline, which limits their range of applications. Here, in order to fabricate eco-friendly electrospun materials, poly(L-lactide-*co*-D,L-lactide) (PLDLLA) was selected as a biodegradable and biocompatible polymer. In particular, this copolymer (composed of 70% L-units and 30% D,L-units) is amorphous with more flexibility and better degradation (hydrolytic or enzymatic) rate compared to the PLA homopolymer [29]. Thus, the present study is the first report on the fabrication of novel, more effective, easy to use and to remove hybrid fibrous materials based on PLDLLA, Fe_3_O_4_ and ZnO with purposely tailored design for antioxidant and photocatalytic performance. The changes in morphology, thermal properties and crystallinity of the hybrid fibrous PLDLLA materials were characterized in detail by SEM equipped with EDX, TEM, TGA and XRD analyses, respectively. The antioxidant activity of the prepared fibrous materials with an appropriate design was tested by the DPPH free radical assay, and their photocatalytic activity and reusability against model organic pollutant methylene blue dye under UV light irradiation were also evaluated.

## 2. Materials and Methods

### 2.1. Materials

Poly(L-lactide-*co*-D,L-lactide) (PLDLLA; Resomer^®^ LR-708, pellets, 910,000 g/mol, Mw/Mn = 2.46; molar ratio L-lactide/D,L-lactide = 69/31) was kindly donated by Boerhinger-Ingelheim Chemicals Inc. (Ingelheim am Rhein, Germany). Commercially available iron oxide (Fe_3_O_4_, nanopowder 20–30 nm, NanoAmor, Houston, TX, USA) and zinc oxide (ZnO, Zano^®^20, Umicore Zinc Chemicals, Olen, Belgium) were used as received. Methylene blue (MB), 2,2-diphenyl-1-picrylhydrazyl free radical (DPPH•), dichloromethane (DCM) and *N*,*N*-dimethylformamide (DMF) were purchased from Sigma-Aldrich (St. Louis, MO, USA). All chemicals were of analytical grade and used without further purification.

The quaternized *N*,*N*,*N*-trimethyl chitosan iodide (QCOS) was synthesized from chitosan oligomer (COS, 10,000 g/mol, Kitto Life Co. LTD, Pyeongtaek, Republic of Korea) as reported previously [30] according to a known procedure [31,32]. The QCOS substitution degrees were determined by ^1^H NMR (600 MHz, in D_2_O) as reported in details elsewhere [30] and were 63% (*N*,*N*,*N*-trimethylation degree), 23% (*N*,*N*-dimethylation degree), 41% (methylation degree of –OH groups at H-3) and 90% (methylation degree of –OH groups at H-6).

### 2.2. Preparation of Electrospun Materials

In the present study, four types of electrospun materials were fabricated. Fibrous PLDLLA (type 1) and Fe_3_O_4_-*in*-PLDLLA (type 2) materials were prepared by electrospinning, whereas ZnO/QCOS-*on*-PLDLLA (type 3) and ZnO/QCOS-*on*-(Fe_3_O_4_-*in*-PLDLLA) (type 4) materials were prepared by simultaneous electrospinning and electrospraying.

For the preparation of all types of materials PLDLLA (5% *w*/*v*) was dissolved in a mixed DCM/DMF (3/1 *v*/*v*) solvent. Type 2 materials (design type “*in*”) were obtained by adding Fe_3_O_4_ (0.5% *w*/*v*) in PLDLLA (5% *w*/*v*) solution. The prepared PLDLLA/Fe_3_O_4_ (10/1) mixture was well dispersed on vortex (V-1 plus, Boeco, Hamburg, Germany) and then sonicated (Bandelin Sonorex, 160/640 W, 35 kHz) for 30 min. The electrospinning was performed for a deposition time of 10 h at an applied voltage of 20 kV, tip-to-collector distance of 20 cm, constant feed rate of 2 mL/h and collector rotation speed of 1500 rpm, as well as constant ambient conditions—room temperature (22 °C) and relative humidity of 51%.

Type 3 and type 4 materials (design type “*on*”) were obtained by simultaneous electrospinning of PLDLLA solution or PLDLLA/Fe_3_O_4_ mixture, and electrospraying of a ZnO/QCOS dispersion. For that purpose, ZnO (1 wt. %) was added under vigorous stirring to a QCOS (0.1% *w*/*v*) solution in water/ethanol (7/3 *v*/*v*). The obtained ZnO/QCOS dispersion was sonicated for 30 min. Subsequently, simultaneous electrospinning and electrospraying were conducted for a deposition time of 10 h at an applied voltage of 20 kV, collector rotation speed of 1500 rpm, constant room temperature (22 °C) and relative humidity of 51%. In this case, an additional pump for delivering the electrospraying dispersion and another high-voltage supply were used. Furthermore, electrospinning was performed under the same conditions as for type 1 and 2 materials, while electrospraying was conducted at a 5 cm tip-to-collector distance and 1 mL/h flow rate of ZnO/QCOS dispersion.

After the fabrication, all types of electrospun materials were dried in a heated vacuum desiccator (Vacuo-Temp, J.P. Selecta, Abrera, Barcelona, Spain) at 30 °C in order to remove all solvent residue, if any.

### 2.3. Characterization

The surface morphology of the prepared electrospun materials was observed by scanning electron microscopy (SEM, JEOL JSM 6390). Specimens were placed on the sample holders and vacuum-coated with gold. The average fiber diameter was evaluated by measuring at least 40 fiber diameters from each SEM image. The distribution of the Fe_3_O_4_ and ZnO particles was observed by transmission electron microscopy operating at 200 kV (TEM, JEM 2100, JEOL Ltd., Tokyo, Japan) and by tabletop SEM (SNE-4500M Plus B) equipped with EDX system (Bruker, Berlin, Germany) for elemental mapping. Specimens were prepared by depositing them on the copper grid and coating them with gold (Ion Sputter Coater, MCM-100P, New Taipei city, Taiwan), respectively. The surface wettability of the fibrous materials also was determined using an Easy Drop DSA20E Krűss drop shape analysis system (Hamburg, Germany). A sessile droplet of distilled water (10 μL) controlled by a computer dosing system was deposited onto the fibrous sample surface and the static water contact angle was calculated by computer analysis of the acquired images of the droplet by DSA1 software (v 1.92-05). The data are averaged from 10 measurements for each sample.

Thermogravimetric analyses (TGA) were carried out on a Perkin Elmer TGA 4000 (Waltham, MA, USA). Measurements were run at a heating rate of 10 °C/min under an argon flow of 60 mL/min to avoid any thermo-oxidative degradation. Instrument control, data acquisition, and data processing were performed by Pyris software (v.11.0.0.0449). Phase composition of the electrospun materials was assessed by X-ray diffraction analysis (XRD). XRD spectra were recorded at room temperature using a D8 Bruker Advance powder diffractometer (Billerica, MA, USA) with a filtered CuKα radiation source and a luminescent detector. The analyses were performed in the 2θ range from 10° to 80° with a step of 0.02° and counting time of 1 s/step. In order to determine the magnetization of fibrous materials, samples were fixed in a quartz holder and placed in the vibrating-sample magnetometer (VSM). Magnetization was measured at 300 K by applying an increasing magnetic field.

### 2.4. Antioxidant Activity

The ability of the fabricated fibrous materials to scavenge radicals was studied with 2,2-diphenyl-1-picrylhydrazyl free radical (DPPH•) assay. In a typical run, electrospun samples with a weight of 0.025 g were cut and mixed with 2 mL of 0.1 mM ethanol solution of DPPH•. For comparison, blank samples of ZnO powder (0.25% *w*/*v*), Fe_3_O_4_ nanopowder (0.125% *w*/*v*) and QCOS (0.025% *w*/*v*) were also mixed with 2 mL of 0.1 mM ethanol solution of DPPH•. Then, all mixtures were incubated in the dark at room temperature for 30 min followed by absorbance measuring at 517 nm with a UV–Vis spectrophotometer (DU 800 Beckman Coulter Inc., Brea, CA, USA). The percentage of DPPH• scavenging activity (AA, %) was calculated by the following equation:

AA, % = [(A_DPPH●_ − A_sample_)/A_DPPH●_] × 100, (1)

where A_DPPH●_ is the absorbance of the DPPH• in ethanol and A_sample_ is the absorbance of the sample. The experiment was run in triplicate.

### 2.5. Photocatalytic Activity and Reusability Test

The photocatalytic activity of the electrospun materials was assessed using UV lamp (400F/2, UVASPOT 400/T, Dr. Hönle AG, Gilching, Germany) for degradation of model organic pollutant—methylene blue (MB). Samples (~90 mg) were cut and immersed in 10 mL of the aqueous solution of MB dye (2 × 10^−5^ mol/L). Prior UV light irradiation samples were kept for 30 min in the dark to attain an adsorption–desorption equilibrium. After that, samples were irradiated under UV light for 3 h. Additionally, MB solution was also irradiated under the same conditions, as a blank control. The intensity decrease in the MB absorbance (at 664 nm) with the irradiation time was monitored spectrophotometrically with a DU800 spectrophotometer (Beckman Coulter Inc., Brea, CA, USA). The reusability of the fibrous hybrid materials was tested by immersing the same samples into a fresh MB solution after each photocatalysis run. This procedure was repeated 5 times for 3 h.

## 3. Results and Discussion

**Scheme 1:**
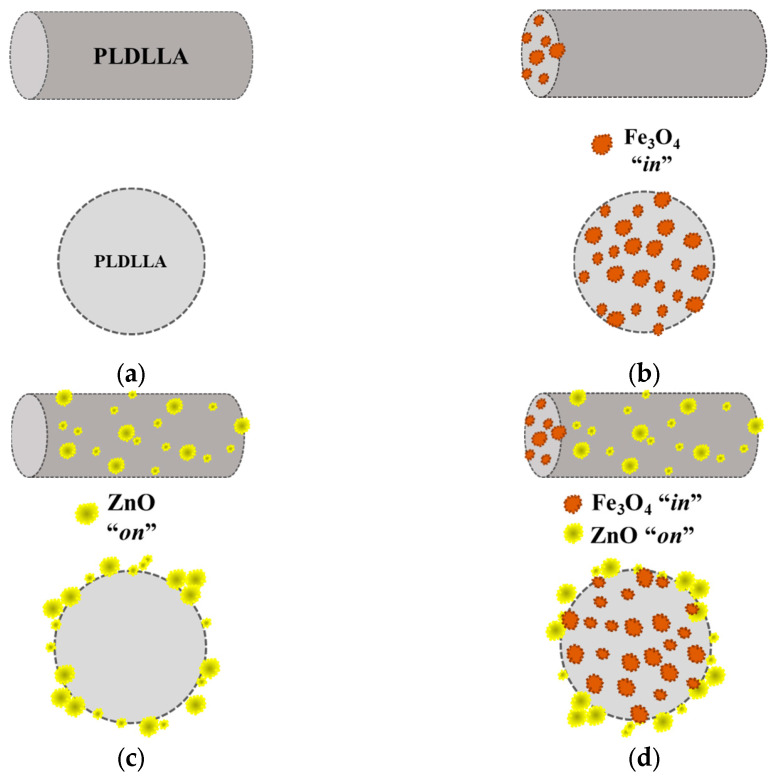
Design types of the fabricated fibrous materials: (**a**) PLDLLA, (**b**) Fe_3_O_4_-*in*-PLDLLA, (**c**) ZnO/QCOS-*on*-PLDLLA and (**d**) ZnO/QCOS-*on*-(Fe_3_O_4_-*in*-PLDLLA).

Herein, with the intention of improving the PLDLLA electrospinnability, a co-solvent system of DCM/DMF (3/1 *v*/*v*) was used. DCM was chosen instead of the widely used chloroform [33] because of its higher vapor pressure, while DMF was added to increase the conductivity of the resultant spinning solution and charge density at the jet, similar to [24,25,26,34]. The effect of the DCM/DMF ratio, PLDLLA concentration and electrospinning conditions (applied voltage, flow rate and tip-to-collector distance) on the PLDLLA fiber morphology was preliminary examined. As a result, the optimal conditions for the formation of a stable Taylor cone required for the fabrication of continuous defect-free PLDLLA fibers were found. To the best of our knowledge, no report has been found to use this co-solvent system. In that way, four types of fibrous materials were prepared (Scheme 1). Based on our previous experience [25], Fe_3_O_4_ was incorporated into PLDLLA fibers, imparting magnetic properties (Scheme 1b,d), whereas dispersion of ZnO particles in the presence of quaternized chitosan derivative (QCOS) was electrosprayed onto the surface of the fibers (Scheme 1c,d), imparting antioxidant and photocatalytic properties. Here, QCOS as a biodegradable, biocompatible and water-soluble polymer with beneficial biological activities [35,36,37] was chosen as a sticking agent for ZnO particles. In addition, the performed preliminary experiments showed that QCOS is an excellent stabilizing agent for dispersions of ZnO particles in water/ethanol (7/3 *v*/*v*). Thus, in one step, by electrospinning of PLDLLA and its mixture with Fe_3_O_4_ (Scheme 1a,b) and by simultaneous electrospinning of PLDLLA or its mixture with Fe_3_O_4_ and electrospraying of ZnO/QCOS dispersion (Scheme 1c,d), fibrous materials with targeted design were developed.

### 3.1. Morphological Characterization of the Electrospun Materials

**Figure 1:**
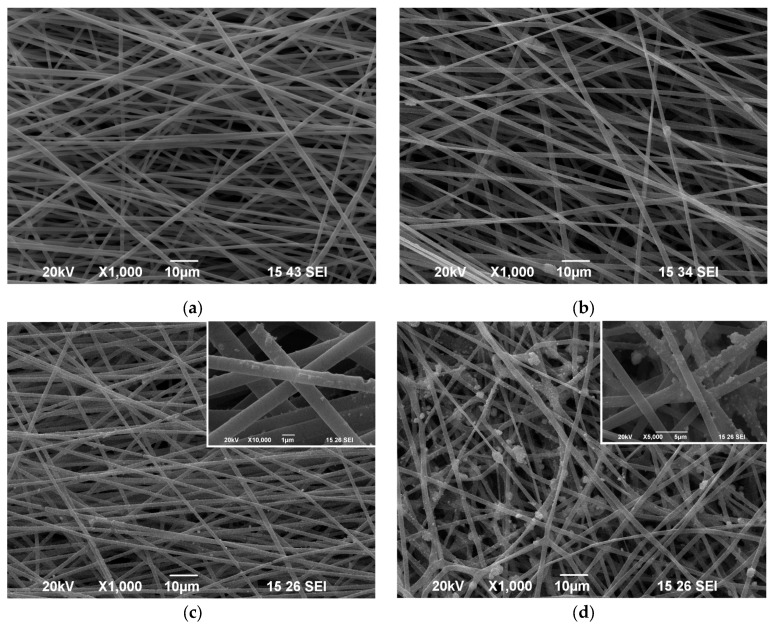
SEM micrographs of the fabricated fibrous materials: (**a**) PLDLLA, (**b**) Fe_3_O_4_-*in*-PLDLLA, (**c**) ZnO/QCOS-*on*-PLDLLA and (**d**) ZnO/QCOS-*on*-(Fe_3_O_4_-*in*-PLDLLA).

Changes in the morphology of the fabricated PLDLLA-based materials were observed by SEM (Figure 1). As expected, electrospinning of PLDLLA (Figure 1a) resulted in monolith, cylindrical and defect-free fiber formation. It is also clearly seen (Figure 1b) that the addition of Fe_3_O_4_ nanopowder to the PLDLLA spinning solution did not change drastically the morphology of the electrospun fibers. Evidently, thicker parts along the fiber length due to the Fe_3_O_4_ incorporation, were observed. Thus, the optimal conditions for defect-free PLDLLA and uniform Fe_3_O_4_-*in*-PLDLLA fiber formation were found. In contrast to the electrospun materials, those fabricated by simultaneous electrospinning and electrospraying show a drastic change in the morphology (Figure 1c,d), with the deposition of ZnO particles or its aggregates on the surface of the fibers and along their length. In that manner, a rough surface typical for design type “*on*” was observed (Figure 1c,d insets). This morphological change can be attributed to the changes in the fabrication approach in agreement with our previous studies [24,25,38]. More importantly, QCOS was added to the ZnO dispersion as an agent with a dual role—stabilizing dispersion and sticking the particles onto the fibers. From the SEM images, the fiber diameters were also measured and the fiber diameter distribution is presented in Figure S1. The average fiber diameters of the PLDLLA and ZnO/QCOS-*on*-PLDLLA were 1.12 µm ± 0.13 µm and 1.30 µm ± 0.17 µm, respectively. Expectedly, those of the Fe_3_O_4_-*in*-PLDLLA and ZnO/QCOS-*on*-(Fe_3_O_4_-*in*-PLDLLA) fibers increased and were 1.52 µm ± 0.25 µm and 1.53 µm ± 0.15 µm, respectively. Apparently, the addition of Fe_3_O_4_ in PLDLLA leads to this slight increase in the average fiber diameters. The obtained SEM results are in agreement with the proposed schematic representation (Scheme 1) and showed that the targeted design types “*in*” and “*on*” can be successfully achieved in one step by a combination of electrospinning and electrospraying.

**Figure 2:**
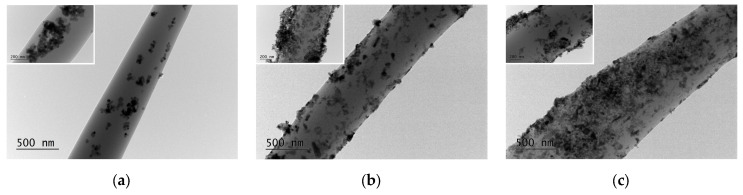
TEM images of the fabricated hybrid fibrous materials: (**a**) Fe_3_O_4_-*in*-PLDLLA, (**b**) ZnO/QCOS-*on*-PLDLLA and (**c**) ZnO/QCOS-*on*-(Fe_3_O_4_-*in*-PLDLLA).

The resultant particle distribution of Fe_3_O_4_ and ZnO was analyzed by TEM (Figure 2). It can be seen that the proper homogenization of the Fe_3_O_4_ particles in the PLDLLA solution results in their almost uniform distribution along the length of the fiber during the electrospinning of the Fe_3_O_4_/PLDLLA mixture (Figure 2a). However, the formation of small aggregates of some of the Fe_3_O_4_ particles is also observed (Figure 2a inset). This outcome can be ascribed to both the rapid evaporation of the co-solvent system during the electrospinning process and the electrostatic attraction between the particles during this process, despite their good dispersion. Conversely, during the simultaneous electrospraying relatively uniform distribution of ZnO particles onto the surface of the fibers was detected (Figure 2b,c). In addition, electrospraying results in the enrichment of the fiber surface with ZnO particles and thus significantly improves its roughness. Apparently, numerous active sites on the fiber’s surface were created, allowing direct interaction with the polluted media. Consequently, by increasing the surface area of the fibers, the capability of pollutant removal is also increased. As clearly seen, electrospraying also leads to the formation of ZnO aggregates on the fiber surface, similar to electrospinning (Figure 2b,c insets). In this case, apart from the rapid evaporation of the solvent, the addition of QCOS also plays a role in the sticking and aggregation of the particles. These results are consistent well with those from SEM analysis and confirm that in one step by simultaneous electrospinning and electrospraying, the targeted design of the fibrous materials might be successfully achieved.

**Figure 3:**
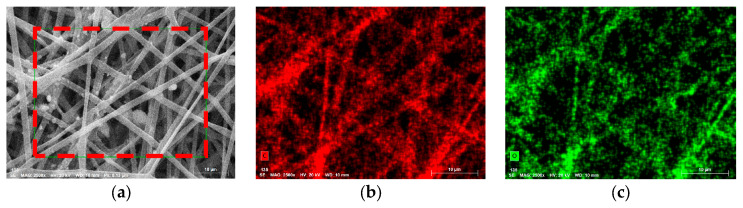

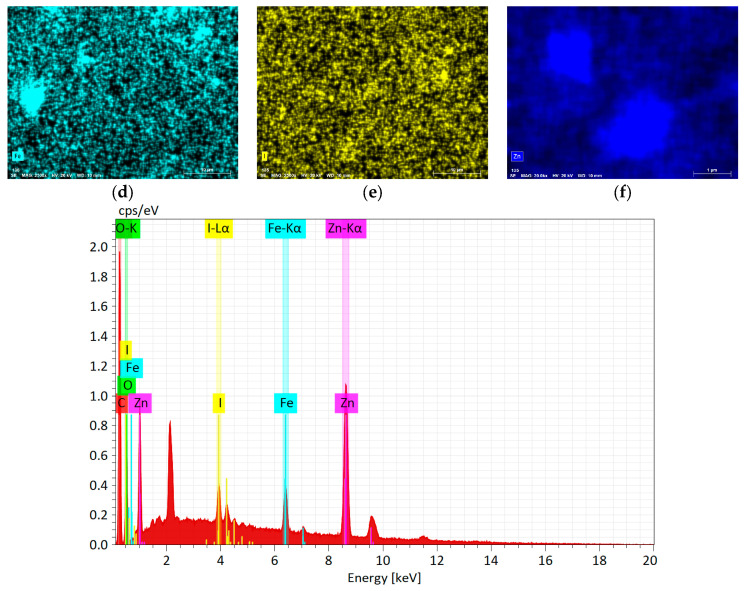
SEM-EDX analysis of the fabricated hybrid fibrous ZnO/QCOS-*on*-(Fe_3_O_4_-*in*-PLDLLA) materials: (**a**) SEM image, EDX elemental mapping of (**b**) carbon, (**c**) oxygen, (**d**) iron, (**e**) iodine, (**f**) zinc and EDX spectrum.

Additional information on the distribution of Fe_3_O_4_ and ZnO particles in the hybrid fibrous ZnO/QCOS-*on*-(Fe_3_O_4_-*in*-PLDLLA) materials was also obtained from the performed energy-dispersive X-ray spectroscopy, as well (Figure 3). EDX elemental mapping (Figure 3b–f) revealed the presence of carbon, oxygen, iron, iodine and zinc, and in agreement with TEM analysis confirmed the positioning of Fe_3_O_4_ and ZnO particles along the fiber length. In general, Fe_3_O_4_ particles are relatively well dispersed and form smaller aggregates (Figure 3d) compared to those of ZnO (Figure 3f). This is probably due to the different processes used for their incorporation—electrospinning and electrospraying—as well as the presence of QCOS (Figure 3e) in the ZnO dispersion.

**Figure 4:**
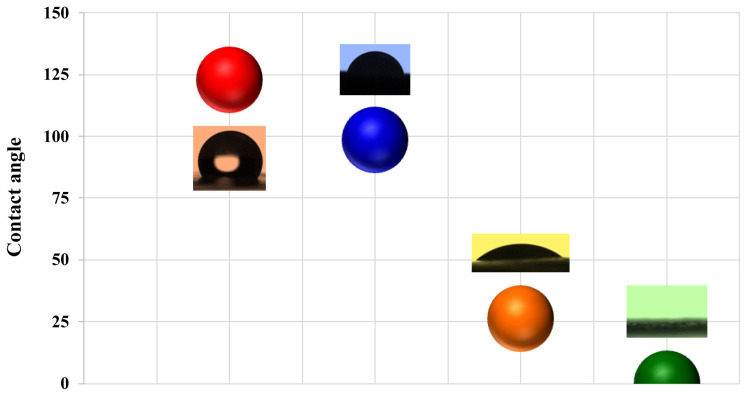
Wettability of the electrospun PLDLLA (red), Fe_3_O_4_-*in*-PLDLLA (blue), ZnO/QCOS-*on*-PLDLLA (orange) and ZnO/QCOS-*on*-(Fe_3_O_4_-*in*-PLDLLA) (green) materials. Representative droplet images are also shown.

Morphological studies proved the presence of ZnO particles on the surface of the PLDLLA and Fe_3_O_4_-*in*-PLDLLA fibers. This purposely tailored surface alternation implies a change in the wettability of the electrospun materials as well. Indeed, the water contact angle (WCA) of the fibrous materials (Figure 4) decreases in the order: PLDLLA (122.9° ± 2.4°) > Fe_3_O_4_-*in*-PLDLLA (98.6° ± 1.7°) > ZnO/QCOS-*on*-PLDLLA (26.2° ± 3.3°) > ZnO/QCOS-*on*-(Fe_3_O_4_-*in*-PLDLLA) (0°). As expected, the WCA of fibrous PLDLLA and Fe_3_O_4_-*in*-PLDLLA materials was larger than 90°, due to the hydrophobic nature of the PLA. In contrast, hybrid fibrous material type “*on*” showed a WCA of less than 90°, which proves their hydrophilicity. In particular, this is due to both the zinc oxide and the QCOS. In that manner, in one step, the surface wettability of electrospun hybrid materials can be easily targeted and modified. Moreover, improved wettability enhances the adsorption ability and thus facilitates the photocatalytic performance of the prepared hybrid materials.

Morphological results are in agreement with the hypothesis presented in Scheme 1 and show that suitable conditions for the fabrication of fibrous materials with design type “*in*” and “*on*” were found. In addition, the improved wettability of the hybrid electrospun materials with purposely tailored design suggests their promising application as antioxidant and photocatalytic agents.

### 3.2. Thermal Behavior and Crystallinity of the Electrospun Materials

Thermogravimetric analysis (TGA) was performed in order to evaluate the effect of the inorganic particles (Fe_3_O_4_ and ZnO) on the thermal properties of PLDLLA fibers. The amount of the embedded inorganic particles was also determined. In that manner, the efficiency of the proposed approach was verified.

**Figure 5:**
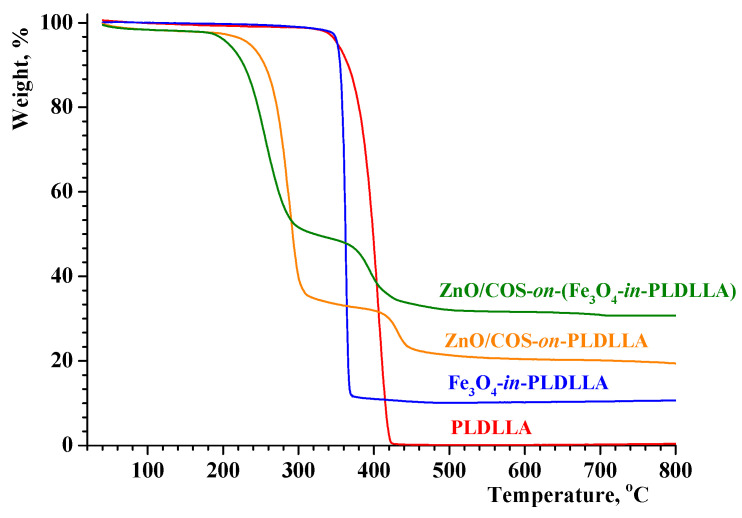
TGA curves of the electrospun PLDLLA, Fe_3_O_4_-*in*-PLDLLA, ZnO/QCOS-*on*-PLDLLA and ZnO/QCOS-*on*-(Fe_3_O_4_-*in*-PLDLLA) materials.

Thermal decomposition of the electrospun materials was studied in the temperature range from room temperature to 800 °C (Figure 5). As seen, PLDLLA and Fe_3_O_4_-*in*-PLDLLA materials undergo a one-stage decomposition, typical for polylactide materials [39,40], which starts at 320 °C and ends at 420 °C with maxima at 408 °C and 363 °C, respectively. PLDLLA fibers decompose entirely with almost 100% weight loss at 800 °C, while hybrid fibrous materials reveal a higher residual mass of 9.9%. This residual mass is much closer to the amount of Fe_3_O_4_ added in the mixed spinning solution and is apparently due to its presence in the PLDLLA fibers as it remains unchanged at these high temperatures as an inorganic component. In contrast, the thermal decomposition of the ZnO/QCOS-*on*-PLDLLA and ZnO/QCOS-*on*-(Fe_3_O_4_-*in*-PLDLLA) materials was carried out in two clearly defined stages. The first stage began at 200 °C and ended at 320 °C with considerable weight losses, while the second stage ended at 480 °C. Probably, in this case, the decomposition stages of PLDLLA and QCOS are overlapped. Evidently, the relevant weight losses were close to the theoretical ones for PLDLLA and QCOS. Furthermore, thermal degradation typical of polylactides in the presence of zinc compounds also occurs, which is consistent with previously reported in the literature [41]. The corresponding derivative thermogravimetric (DTG) curves are given in Figure S2. It is noteworthy that the decomposition of the ZnO/QCOS-*on*-PLDLLA ended with 18.2% residue, while this of the ZnO/QCOS-*on*-(Fe_3_O_4_-in-PLDLLA) materials with 28.1%. These residues might be ascribed to the ZnO and Fe_3_O_4_ because their amounts are very close to the initial amounts of the ZnO added to the electrosprayed dispersion and to the Fe_3_O_4_ in the mixed spinning solution. In addition, the thermal decomposition of the ZnO and Fe_3_O_4_ proceeds without destruction over 800 °C. The observed decrease in the thermal stability of PLDLLA in the hybrid fibrous material type “*on*” is probably due to both the known poor thermal stability of QCOS and the presence of ZnO [41,42].

**Figure 6:**
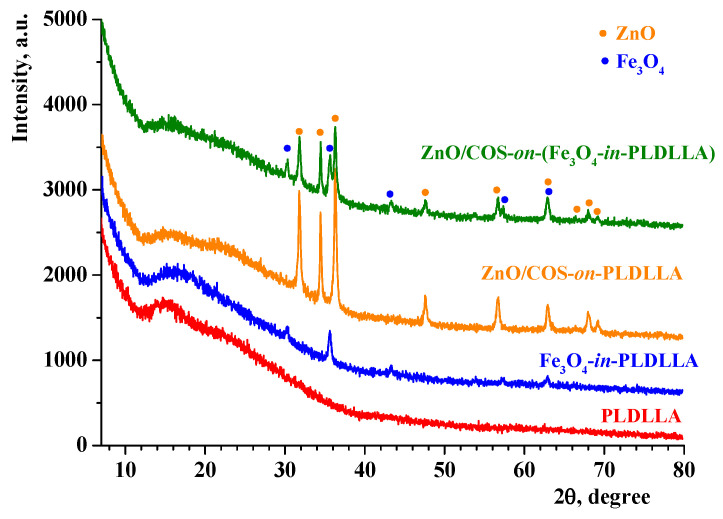
XRD patterns of the electrospun PLDLLA, Fe_3_O_4_-*in*-PLDLLA, ZnO/QCOS-*on*-PLDLLA and ZnO/QCOS-*on*-(Fe_3_O_4_-*in*-PLDLLA) materials.

In addition, the phase structures of the electrospun materials were analyzed by X-ray diffraction (XRD). In the range of small angles (Figure 6), the amorphous halo for all materials is associated with the amorphous structure of PLDLLA. In particular, the rapid solidification of the fibers during the electrospinning process limits the spatial arrangement of the stretched polymer chains into ordered crystal structures. All patterns in the XRD of the hybrid Fe_3_O_4_-*in*-PLDLLA, ZnO/QCOS-*on*-PLDLLA and ZnO/QCOS-*on*-(Fe_3_O_4_-*in*-PLDLLA) materials confirm the presence of the cubic phase of magnetite (PDF 89-0691) and hexagonal phase of zinc oxide (PDF 36-1451). The Fe_3_O_4_ crystal phase was identified with peaks at 30.3 (220), 35.6 (311), 43.3 (400), 57.1 (511) and 62.8 (440), while that of ZnO had peaks at 31.8 (100), 34.5 (002), 36.3 (101), 47.6 (102), 56.7 (110), 62.9 (103), 66.4 (200), 67.9 (112) and 69.2 (201). These clearly defined reflexes indicated that the incorporation of Fe_3_O_4_ in the fibers and ZnO onto the fibers’ surface did not have any impact on the amorphous structure of the PLDLLA. Moreover, the electrospinning/electrospraying processes did not affect the crystallinity of the inorganic particles.

Imparting magnetic properties of the fibrous materials by adding Fe_3_O_4_ is an alternative for their rapid separation during subsequent operation by applying an external magnetic field. For that reason, the saturated magnetization of the hybrid fibrous materials was measured by using of vibrating sample magnetometer (VSM). The Fe_3_O_4_-*in*-PLDLLA materials showed better magnetization (2.7 emu/g) than the ZnO/QCOS-*on*-(Fe_3_O_4_-*in*-PLDLLA) materials (below 1 emu/g), possibly due to the lower amount magnetic material included in them. Therefore, the incorporation in the PLDLLA fiber amount of Fe_3_O_4_ was sufficient for rapid separation of the electrospun materials under the action of a permanent magnet.

### 3.3. Antioxidant Activity

**Figure 7:**
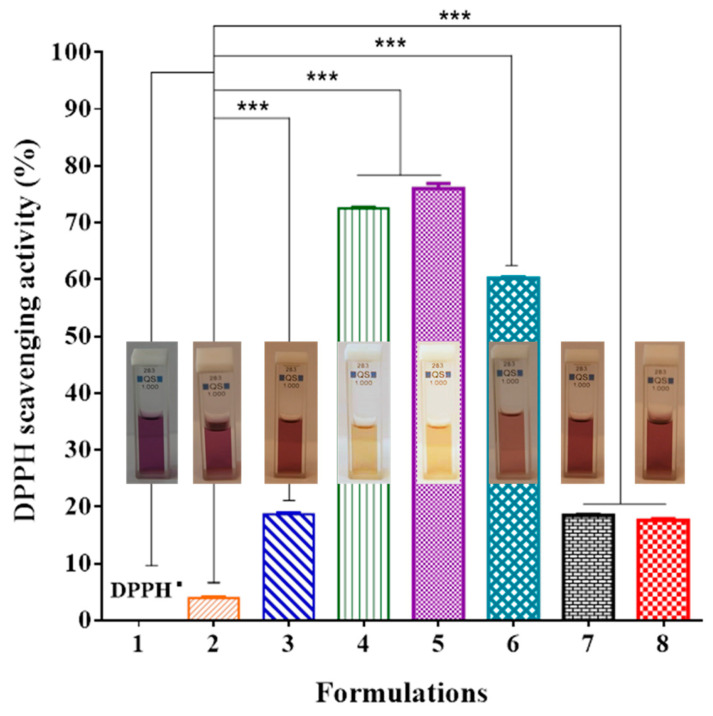
DPPH radical scavenging activity of the electrospun (2) PLDLLA, (3) Fe_3_O_4_-*in*-PLDLLA, (4) ZnO/QCOS-*on*-PLDLLA and (5) ZnO/QCOS-*on*-(Fe_3_O_4_-*in*-PLDLLA) materials. Controls: (1) DPPH solution, (6) ZnO, (7) Fe_3_O_4_ and (8) QCOS. *** *p* < 0.001. Digital photographs of the corresponding solutions are shown as well.

As ZnO and QCOS possess antioxidant activity [35,42,43,44], the DPPH radical scavenging assay was used to evaluate the antioxidant potential of the prepared electrospun materials. In general, materials with design type “*on*” displayed the highest antioxidant activity compared to that of material type “*in*” (Figure 7). Evidently, within 30 min of contact, ZnO/QCOS-*on*-(Fe_3_O_4_-*in*-PLDLLA) and ZnO/QCOS-*on*-PLDLLA materials showed 76.0 ± 0.8% and 72.5 ± 0.2% DPPH• scavenging capacity, respectively. As seen in Figure 7, ZnO exhibited higher free radical scavenging activity (60.2 ± 0.2%) than both Fe_3_O_4_ (18.5 ± 0.1%) and QCOS (17.6 ± 0.3%). Conversely, fibrous PLDLLA materials have the lowest antioxidant activity (3.9 ± 0.2%). However, the incorporation of Fe_3_O_4_ into PLDLLA fibers leads to a slight increase in their antioxidant activity and it becomes close to that of pristine Fe_3_O_4_ (Figure 7). Probably, deposited on the surface of the fiber ZnO particles act as electron acceptors and thus quench the DPPH radicals. Apparently, the addition of QCOS has a synergistic effect on the action of ZnO leading to an increase in the overall antioxidant activity, thus significantly improving the performance of the fibrous materials with design type “*on*”.

### 3.4. Photocatalytic Activity and Reusability

Besides the high surface area, the wettability, thermal and magnetic properties, and antioxidant activity of the fabricated electrospun hybrid materials demonstrate their great potential as eco-friendly filtering membranes in long-term large-scale water purification. Moreover, the purposely tailored design of the fibrous materials and the high photocatalytic activity of ZnO against organic pollutants suggested their excellent photocatalytic performance. Among the organic dyes, methylene blue (MB) is toxic, carcinogenic and non-biodegradable, and is one of the most abundant organic pollutants in water. For that reason, the photocatalytic activity of the obtained fibrous materials under UV light irradiation against MB as a model organic pollutant was evaluated by monitoring the reaction of discoloration at 660 nm by recording the decrease in the MB absorption as a function of irradiation time.

**Figure 8:**
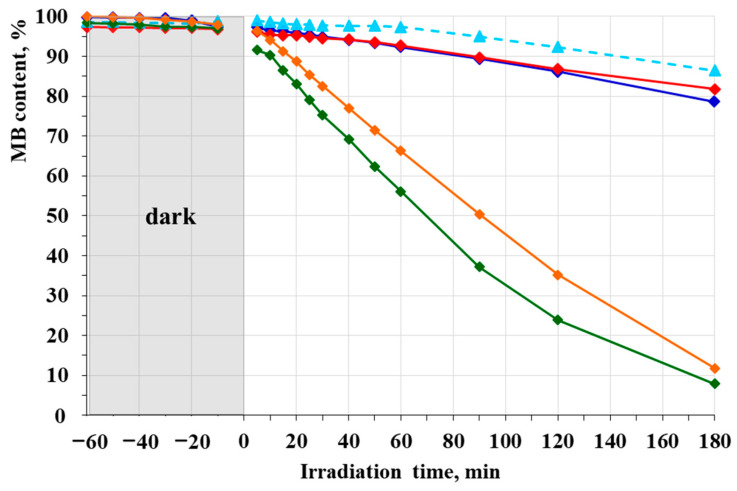
Photocatalytic degradation of MB at 660 nm in the presence of fibrous PLDLLA (red line) and hybrid fibrous Fe_3_O_4_-*in*-PLDLLA (dark blue line), ZnO/QCOS-*on*-PLDLLA (orange line) and ZnO/QCOS-*on*-(Fe_3_O_4_-*in*-PLDLLA) (green line) materials. Blank sample of MB solution (light blue dashed line).

The dependence of MB photocatalytic degradation on UV irradiation time for fibrous materials is shown in Figure 8. As expected, after 3 h of UV irradiation only 13% of MB (blank sample) was degraded, while in the presence of PLDLLA fibers and fibrous Fe_3_O_4_-*in*-PLDLLA materials, the MB degradation was 18% and 21%, respectively. These results indicate that under UV light irradiation self-degradation of MB occurs, but at a very slow rate. Probably, the low degree of dye degradation in the presence of materials with design type “*in*” is due to its adsorption. Noteworthy, the degradation of MB in the dark for 1 h in the presence of fibrous materials was similar to that of the blank sample (below 5%), implying that the absorption of MB on the fibrous materials was limited after reaching the adsorption–desorption equilibrium. Apparently, under UV irradiation in the presence of fibrous ZnO/QCOS-*on*-PLDLLA and ZnO/QCOS-*on*-(Fe_3_O_4_-*in*-PLDLLA) materials, 50% of MB was degraded for 70 and 90 min. In addition, after 3 h of UV irradiation, these materials degraded 88% and 92% of MB, respectively. Therefore, almost complete MB degradation occurred in the presence of materials with design type “*on*”. This indicates that the deposition of ZnO photocatalyst on the fiber’s surface significantly enhanced the photocatalytic activity of the fibrous materials and preserved its activity after electrospraying.

**Figure 9:**
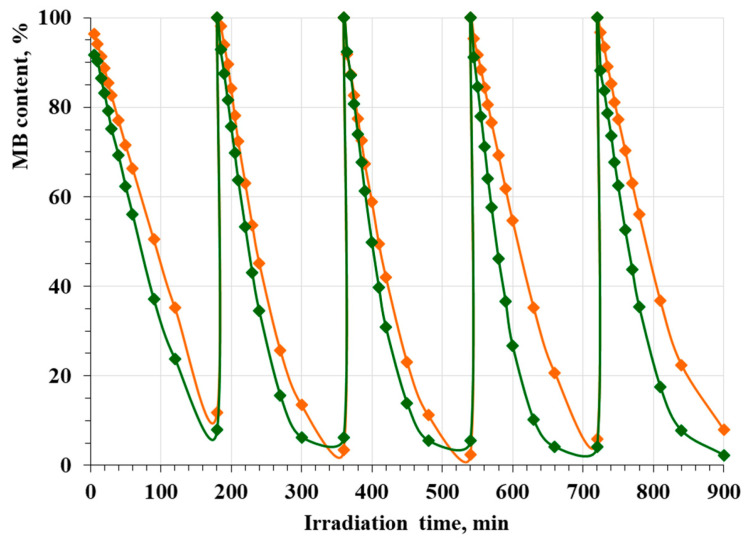
Photocatalytic degradation of MB during five consecutive photooxidation cycles in the presence of ZnO/QCOS-*on*-PLDLLA (orange line) and ZnO/QCOS-*on*-(Fe_3_O_4_-*in*-PLDLLA) (green line) materials.

The reusability of the fibrous materials, as one of the most important criteria for evaluating the feasibility of large-scale applications, was also determined by measuring their photoactivity during five irradiation cycles. Clearly, fibrous ZnO/QCOS-*on*-PLDLLA and ZnO/QCOS-*on*-(Fe_3_O_4_-*in*-PLDLLA) materials show excellent reusability in five subsequent irradiation cycles (Figure 9). It is evident that after fivefold use materials with design type “*on*” exhibit excellent stability and preserve almost completely their photocatalytic activity. Moreover, at the end of the fifth cycle, these materials degraded more than 90% of MB. Notably, after the reusability test the hybrid fibrous materials do not disintegrate, preserve specimen shape and retain their fibrous structure, and also the weight losses were negligible.

## 4. Conclusions

A facile and effective one-step approach for the successful fabrication of electrospun effective and eco-friendly hybrid materials based on PLDLLA was proposed. In order to impart magnetic properties, Fe_3_O_4_ particles were appropriately incorporated in the PLDLLA fibers, while to impart antioxidant and photocatalytic properties, ZnO particles were deposited on the fiber’s surface. It was shown that these particles had an impact on the morphology, wettability, thermal properties and crystallinity of the fibrous PLDLLA materials. The incorporation in the PLDLLA fiber amount of Fe_3_O_4_ was sufficient for rapid separation of the electrospun materials under the action of a permanent magnet. The presence of ZnO particles on the fiber’s surface improved wettability, but decreased the thermal stability of material type “*on*”. In particular, the incorporation of Fe_3_O_4_ in the fibers and ZnO onto the fiber’s surface did not have any impact on the amorphous structure of the PLDLLA. Without any doubt, the purposely tailored design type “*on*” provided improved antioxidant activity with more than 70% of DPPH• scavenging capacity after 30 min of contact. These materials also exhibited excellent stability and supplied photocatalytic degradation of model organic pollutant MB under UV light irradiation even after 5-fold use. Moreover, at the end of the fifth cycle, these materials degraded more than 90% of MB. Therefore, the prepared electrospun hybrid materials based on PLDLLA are promising for water purification from organic pollutants and dyes by heterogeneous photocatalysis.

## Data Availability

The original contributions presented in the study are included in the article, further inquiries can be directed to the corresponding author.

## Supplementary Materials

The following supporting information can be downloaded at: https://www.mdpi.com/article/10.3390/polym16131814/s1, Figure S1: Fibers diameter distribution of the electrospun materials: (a) PLDLLA, (b) Fe_3_O_4_-*in*-PLDLLA, (c) ZnO/QCOS-*on*-PLDLLA and (d) ZnO/QCOS-*on*-(Fe_3_O_4_-*in*-PLDLLA); Figure S2: DTG curves of the electrospun PLDLLA, Fe_3_O_4_-*in*-PLDLLA, ZnO/QCOS-*on*-PLDLLA and ZnO/QCOS-*on*-(Fe_3_O_4_-*in*-PLDLLA) materials.
