# Supplementary material for: Imparting Photocatalytic and Antioxidant Properties to Electrospun Poly(L-lactide-co-D,L-lactide) Materials
Source: Polymers (Basel). 2024 Jun 26;16(13):1814. doi: 10.3390/polym16131814 (PMC11244129; doi:10.3390/polym16131814)
Supplement: Supplementary file 1 [file polymers-16-01814-s001.zip › polymers-3037695-supplementary.pdf]

polymers

MDPI

Supplementary data

Imparting Photocatalytic And Antioxidant Properties To Electrospun Poly(L-lactide-*co*-D,L-Lactide) Materials

Ina Anastasova, Petya Tsekova, Milena Ignatova and Olya Stoilova *

Laboratory of Bioactive Polymers, Institute of Polymers, Bulgarian Academy of Sciences, Akad. G. Bonchev St, bl. 103A, 1113 Sofia, Bulgaria; stoilova@polymer.bas.bg

* Correspondence: stoilova@polymer.bas.bg (O.S.)

Received: date; Accepted: date; Published: date

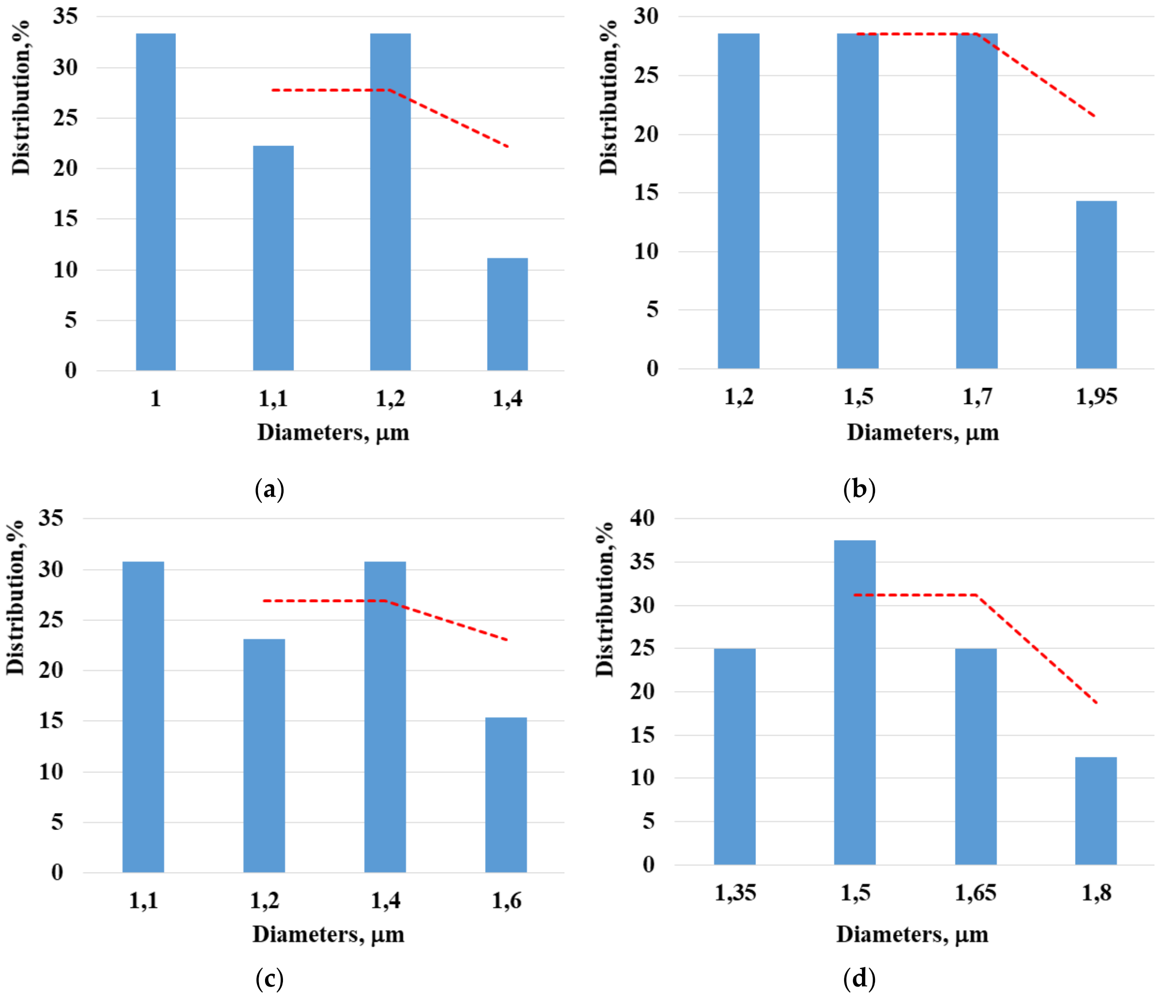

Figure S1. Fibers diameter distribution of the electrospun materials: (**a**) PLDLLA, (**b**) Fe_3O_4-*in*-PLDLLA, (**c**) ZnO/QCOS-*on*-PLDLLA and (**d**) ZnO/QCOS-*on*-(Fe_3O_4-*in*-PLDLLA).

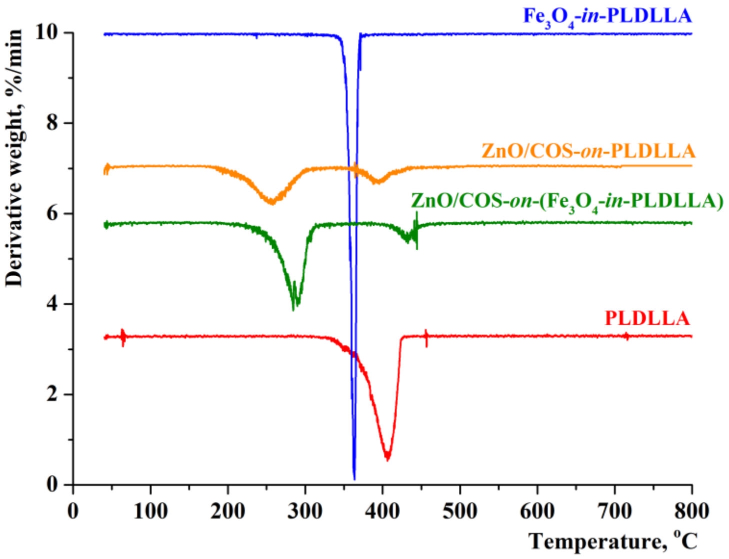

Figure S2. DTG curves of the electrospun PLDLLA, Fe_3O_4-*in*-PLDLLA, ZnO/QCOS-*on*-PLDLLA and ZnO/QCOS-*on*-(Fe_3O_4-*in*-PLDLLA) materials.
